# Transcriptional control of Flt3 ligand targeted by fluorouracil-induced Egr-1 promoter in hematopoietic damage

**DOI:** 10.1186/1423-0127-16-85

**Published:** 2009-09-21

**Authors:** Nan Du, Xuetao Pei, Jinming Zhou, Hui Zhao, Xiaosong Li, Yan Fu, Yixin Hao

**Affiliations:** 1 Department of Oncology First Affiliated hospital, Chinese PLA Postgraduate Medical School, Beijing 100037, China; 2 Department of Stem Cell Biology, Beijing Institute of Transfusion Medicine, Beijing 100850, China

## Abstract

**Background:**

Ionizing radiation (IR) activate the early growth response-1 (Egr-1) promoter by production of radical oxygen intermediates (ROIs). Egr-EF, an expression vector pCIneo containing Egr-1 promoter cloned upstream of the cDNA for Flt3 ligand, was used to treat hematopoietic damage. 5-fluorouracil, a commonly used chemotherapeutic agent, cause tumor cell death by producing DNA damage and generating ROIs. We therefore hypothesized that clinically employed chemotherapeutic agents that increase ROIs could also be employed to activate Egr-EF in a chemoinducible gene therapy strategy. The goal of this study was to explore the effect of Flt3 Ligand gene transcription regulated by fluorouracil-induced Egr-1 promoter on hematopoietic recovery.

**Methods:**

Human Flt3 Ligand (FL) cDNA and enhanced green fluorescent protein (EGFP) cDNA were linked together with IRES and inserted into the expression vector pCI-neo under control of the Egr-1 promoter (Egr-EF). The vector was transfected into the HFCL human bone marrow stromal cell line, and these cells were exposed to 5-FU, a chemotherapeutic drug. Expression of FL by HFCL/EF cells after 5-FU treatment was determined with ELISA, western blot and RT-PCR assays. In addition, the effect of FL from HFCL/EF cell culture supernatants on growth of CD34^+ ^cells from cord blood was also studied. HFCL/EF cells were injected into CB-17 combined immunodeficient (SCID) mice with B16 melanoma. 5-FU was given three days after injection of the HFCL/EF cells. In the recipient mice, white blood cell levels in peripheral blood and expression of EGFP and FL in human stromal cells were measured. Tumor volumes in tumor-bearing mice were also measured.

**Results:**

5-FU treatment increased EGFP levels and secreted FL levels in HFCL/EF cells. Supernatants from HFCL/EF cell cultures treated with 5-FU increased CD34^+ ^cell growth significantly. HFCL/EF exhibited an increase in the number of white blood cells after chemotherapy.

**Conclusion:**

The data presented here support the use of transcriptional control mediated by chemoinducible gene therapy to reduce hematopoietic injury associated with 5-FU.

## Background

Gene therapy for hematopoietic deficiencies is based on delivery and expression of hematopoietic tissue specific genes encoding hematopoietic growth factors [1]. Therapeutic gene delivery systems include vectors, lipofection, naked DNA and cellular vehicles. Poor specificity of hematopoietic tissue targeting and limited control of hematopoiesis-specific gene expression have limited the clinical utility of gene therapy for hematological diseases [2]. One approach to control gene expression is through the use of tissue-selective promoters to activate transcription of transgenes [3]. Another more interesting approach is the eukaryotic inducible expression system [4,5]. It is important to determine whether spatial and temporal control of hematopoietic gene expression following chemotherapy increases the efficiency and safety of radio/chemoinducible gene therapy. Recently, ionizing radiation (IR) has been used to activate target genes for therapy [6]. Cells exposed to IR generate reactive oxygen intermediates (ROIs) that activate radio-inducible CArG [CC (A/T)6GG] DNA elements of the early growth response gene-1 (Egr-1) [7]. Egr-1 expression induces expression of downstream target genes. Therefore, therapeutic genes can be inserted downstream of Egr-1 and their expression can be induced by IR. This approach is known as radiation-gene therapy [8]. This strategy has been used with cDNA encoding human recombinant Flt3 Ligand (FL) that has been ligated into a eukaryotic expression vector. Using this vector system, FL has been preferentially activated in the hematopoietic microenvironment by IR [9]. FL is a cytokine that has been shown to induce early hematopoietic activity in animal studies and is secreted by bone marrow stromal cells and other hematopoietic cells [10]. The combination of FL with chemotherapeutic agents that damage DNA, such as 5-FU and adriamycin, has resulted in synergistic anticancer effects and reduced hematopoietic cytotoxicity in experimental models [11,12].

Similar to IR, chemotherapeutic agents such as 5-FU cause ROI generation and DNA damage. Therefore, chemotherapy can also be used to activate the Egr-1 promoter and induce expression of downstream therapeutic genes. This approach is known as chemo-inducible gene therapy [13]. Although cytokines that stimulate hematopoiesis often result in hematopoietic recovery after chemotherapy, no correlation has been established between expression of chemo-inducible genes and hematopoietic protective effects. A common feature of chemotherapeutic agents is the production of oxygen and other free radical species that lead to DNA damage, lipid peroxidation, protein modification and cellular death [14,15]. ROIs are generated by a number of widely used anticancer drugs including doxorubicin [16], cisplatin [17], cyclophosphamide [18], 5-fluorouracil (5-FU) [19], gemcitabine [20], paclitaxel [21], temozolomide [22] and resveratrol [23]. We hypothesized that ROI generating chemotherapeutic agents could induce production of hematopoietic growth factor in bone marrow stromal cells transfected with a vector encoding the CArG elements of the Egr-1 promoter ligated upstream of cDNA encoding Flt3 Ligand. Fms-like tyrosine kinase 3 (FLT3) ligand (FL) is a novel hematopoietic cytokine that is involved in regulation of early hematopoiesis [24]. Either alone or in combination with other growth factors, FL stimulates the proliferation of highly enriched human and murine hematopoietic stem cells *in vitro *and leads to the proliferation and mobilization of hematopoietic and lymphoid progenitor cells *in vivo *in animals and humans [10]. In addition, FL may enhance dendritic cell (DC)-driven homeostatic T cell expansion and may also improve thymopoiesis [25]. Locally expressed FL may also show anti-tumor effects [11]. Daily subcutaneous administration of FL is associated with dose-limiting systemic side effects. As an alternative to daily systemic subcutaneous injections, localized FL expression restricted to stromal cells within bone marrow may facilitate hematopoietic effects without the systemic toxicity associated with gene transfer [26]. FLT3 gene mutations have been identified as prognostic factors in myeloid malignancies, even though no evidence for constitutive activation of FLT3/FLT3L has been found in such malignancies [27,28]. It is important to determine whether spatial and temporal control of hematopoietic gene expression following chemotherapy increases the efficiency and safety of gene therapy. For example, 5-FU has been replaced in an established 5-Fu combination chemotherapy with the gene therapy/chemotherapy system, Ad-LpCDIRESE1A/5-fluorocytosine (5-FC), in order to reduce toxicity and increase efficacy. This approach is known as "genetic combination therapy" [12]. The goal of using this vector is to decrease the toxic effects of chemotherapy on normal cells and to increase the efficacy of chemotherapy in cancer cells. Using this approach, the concentration of 5-FU administered can be sufficiently high to kill even nondividing cancer cells.

In this study, to decrease systemic toxicity and increase safety of FL, regional delivery approaches were developed to restrict FL to bone marrow stromal cells in the hematopoietic microenvironment. Based on our previous study on Egr-1 promoter regulated Flt3 ligand (FL) or GM-CSF expression induced by ionizing radiation (IR) or chemotherapy [9,29], we report here that 5-fluorouracil (5-FU), a commonly used chemotherapeutic agent that stimulates ROIs generation, induces the production of FL in human bone stromal cells transfected with Egr-EF containing CArG elements cloned upstream of the cDNA for human recombinant FL. 5-FU was used to recover hematopoiesis from chemotherapy- induced marrow failures and CFU-GM in culture and as xenografts in tumor-bearing SCID mice. These data support the use of chemoinducible gene therapy to reduce toxicity of 5-FU and facilitate the effect of the 5-FU-induced FL on hematopoietic recovery after exposure to 5-FU.

## Methods

### Recombinant vector construction

The Egr-EF vector was constructed as follows. FL cDNA and EGFP cDNA were linked together with IRES in the 5' non-translated region and then inserted into the eukaryotic expression vector pCI-neo under the control of the radiation-inducible Egr-1 promoter as described previously [9]. The pCI-F vector (containing FL cDNA without Egr-1 cDNA) and pCI-neo vector (without FL cDNA or Egr-1 cDNA) were used as control vectors. Vectors were stored at -80°C and diluted in formulation buffer (GenVec) to the appropriate concentration before use.

### Cell culture and transfection

Egr-EF, pCI-F and pCI-neo vectors were transfected into human bone marrow stromal cells (human fibroblast cell line, HFCL, ATCC, Rockville, MD, USA) using Lipofectamine (Invitrogen, Carlsbad, CA, USA) according to the manufacturer's instructions as previously described [30]. Positive clones were identified by G418 resistance. A standard clone selection method was used to assay the transduction efficiency. B16 cells (ATCC, Rockville, MD, USA), un-transfected HFCL cells and transfected HFCL cells were maintained in Dulbecco's modified Eagle's medium (DMEM; Gibco BRL, Grand Island, NY, USA) with 10% fetal calf serum (FCS) at 37°C and 5% CO_2_.

Cord blood samples (CB) were obtained from umbilical tissues of full-term deliveries with informed consent of the mothers and used in accordance with procedures approved by the Academy of Military Medical Science of China on Clinical Investigation [31,32]. Mononuclear cells (MNC) were isolated using Ficoll-Hypaque (1.077 ± 0.001 Kg/L, Sigma, St. Louis, MO), washed, and resuspended in Iscove's modified Dulbecco's medium (IMDM; HyClone, Logan, UT) supplemented with 100 mL/L fetal bovine serum (FBS; GibcoBRL, GrandIsland, NY). CD34^+^-enriched cell purification utilized positive selection using the miniMACS immunomagnetic separation system (Miltenyi Biotec Bergish, Glodbach, Germany) according to the manufacturer's instructions as previously described [33]. The purity of selected cord blood CD34^+ ^cells was always greater than 87%, and the cells were cryopreserved in liquid nitrogen until use.

### In vivo studies

Six- to eight-week-old female B17 SCID mice (20 ± 2 g) were purchased from the Laboratory Animal Center of Academy of Military Medical Science (Beijing, China). All animals were housed under specified pathogen free (SPF) conditions. Experiments were performed in accordance with the guidelines of the Institutional Animal Care and Use Committee of the Academy of Military Medical Science of China. The mice were subcutaneously inoculated in the oxter of the left forelimb with 0.2 ml of 2 × 10^6^/ml B16 cells. Five to six days later, healthy mice with tumors were randomly divided into four groups (n = 9/group). Next, 10^6 ^cells (HFCL, HFCL/pCI, HFCL/F, or HFCL/EF cells) were injected into the tail vein of the mice. Three days after injection of the transfected cells, mice were given intraperitoneal injections of 100 mg/kg 5-FU, d1-3. White blood cell counts in the peripheral blood were determined before 5-FU treatment (day 0) and 5, 10, 15, 20, and 25 days after 5-FU treatment. In a second experiment, tumor-bearing mice were randomly divided into four groups (n = 6/group): HFCL/EG + 5-FU, HFCL + 5-FU, HFCL/EG + normal saline (NS), HFCL + NS. The mice were given an intravenous injection of 1 × 10^6 ^transfected cells. After three days, mice were given intraperitoneal injections of 100 mg/kg 5-FU or an equivalent volume of normal saline. Bone marrow cells were harvested from three mice from each group 72 h after injection of 5-FU or saline.

Total RNA was isolated using TRIzol reagent (Invitrogen Life Technologies, Carlsbad, California, USA) from cultured HFCL/EF cells and bone marrow cells of tumor-bearing mice 72 h after 100 mmol/L and 100 mg/kg 5-FU treatment, respectively.

### Blood cell type determination and tumor volume determination

Peripheral blood was harvested from the transfected, tumor-bearing mice at 0, 5, 10, 15, 20 and 25 days after 5-FU or saline injection. The number of each cell type was determined using an automated blood cell counter (NE-8000, Toa. Medical Electronics, Kobe, Japan).

Tumor growth was monitored by periodic measurement with calipers, and tumor

volume, V_t_, was calculated using the following formula: V_t _= 1/2(maximal length, a) × (perpendicular width, b)^2^. The tumor inhibition value, T_I_, was calculated using the following formula: T_I _(%) = [V_t, control group _- V_t, treated group_/V_t, control group_] × 100%. Normalized tumor volumes, V_N_, were calculated using the following formula: V_N _*= *V_t_/V_t, day 0_. Day 0 is the day of vector injection.

### Flow cytometry analysis of enhanced green fluorescent protein reporter

In total, 2 × 10^5 ^HFCL/EF cells were seeded in a 12-well plate. 12 h before treatment, the medium was replaced with serum-free medium. Serum-free medium was used because serum factors have been shown to stimulate the Egr-1 regulatory sequence and induce gene expression. The cells were treated with various concentration of 5-FU (0, 1, 2, 50, 100, 200, 300 and 400 mmol/L) for 8 h, fixed in 10% paraformaldehyde and imaged with a fluorescence microscope (Olympus, Tokyo, Japan). EGFP expression was further determined by FACS as described previously [9]. Bone marrow cells were isolated from the femur of transfected, tumor-bearing mice 3 days after chemotherapy. EGFP-expressing cells were counted by FACS.

### In vitro measurement of FL protein expression

In total, 2 × 10^5 ^HFCL/EF, HFCL/F, HFCL/pCI and HFCL cells were seeded in a

96-well plate (n = 3 wells/group). The cells were allowed to adhere to the plate and were then washed with phosphate buffered saline (PBS) 3 times. The cells in each group were treated with 100 mmol/L 5-FU in serum-free medium. The culture supernatant was harvested at various time points, and human FL expression was detected using an FL ELISA kit (R&D Systems Inc, Minneapolis, MN, USA).

### Effects of HFCL/EF supernatant on CD34^+^ cells

Confluent HFCL/EF cells were treated with 5-FU (100 mmol/L) for 24 h. The medium was replaced with serum-free medium and the cells were incubated for 24 h. Supernatants were then collected. CD34^+ ^cells (1 × 10^4 ^cells/well, 6 wells per group) were cultured in 24-well plates with 30% (v/v) supernatant from transfected 5-FU treated cells, 2-mercaptoethanol (10^4 ^mol/L), hydrocortisone (10^6 ^mol/L), or SCF (50 ng/ml) + IL-3 (20 ng/ml) + IL-6 (20 ng/ml) in serum-free medium (CellGro SCGM, Boehringer Mannheim). Supernatant from HFCL/EF cells that were not exposed to 5-FU was used as a negative control. Supernatants from HFCL/F, HFCL/pCI and HFCL cell cultures 24 h after exposure to 5-FU were also used as negative controls. Cultures were incubated at 37°C with 5% CO_2 _for 10 days before FACS analysis. Two-color flow cytometry was performed on a FacScan (Becton-Dickinson, Mountain View, CA, USA) as previously described [30]. Briefly, cells were incubated in the presence of saturating amounts of monoclonal anti-CD34-FITC (HPCA-1; Becton-Dickinson, Mountain View, CA, USA) and anti-CD38-PE (Leu-17; Becton-Dickinson, Mountain View, CA, USA) antibodies. IgG1 isotype controls conjugated to FITC and PE were also included.

### RT-PCR analysis of FL RNA transcripts

Total RNA in each group was isolated using TRIzol reagent (Invitrogen Life Technologies, Carlsbad, California, USA) from cultured HFCL/EF cells 72 h after 100 mmol/L 5-FU treatment and bone marrow cells from tumor-bearing mice 72 h after 100 mg/kg 5-FU treatment. The following primers were used: FL: P1 5'-GCG GAT CCG CTG GAG GAT GTG GCTG-3'; P2 5'-ATG AAA CAA GAG CTA GAA ACT CAGG-3'; β-actin: P1 5'-AAG GCC AAC CGC GAG AAG AT-3'; P2 5'-TCG GTG AGG ATC TTC ATG GAG-3'. PCR was performed using an RT-PCR kit (Takara, Dalian, China). The PCR conditions were as follows: pre-denaturing at 94°C for 5 min, 36 cycles at 94°C for 60 s, 68°C for 60 s and 72°C for 120 s. PCR products were separated by electrophoresis on a 1.5% agarose gel followed by ethidium bromide staining. The target bands were analyzed by densitometry using a Gel Imaging System (Bio-Rad). The results were calculated as a ratio of OD values for FL and β-actin relative to mouse GAPDH (gel control).

### Western blot analysis for the expression of FL protein in HFCL/EF

Transfected cultured cells and bone marrow cells for each group were harvested after 72 h treatment with 5-FU. Aliquots of cell lysates (50 μg protein) were separated on a 12% NuPAGE gel (Invitrogen) and transferred to nitrocellulose filters. The filters were blocked with TBST buffer containing 5% skimmed milk and incubated with FL monoclonal antibodies (1:2000, Calbiochem, Cambridge, MA, USA) and GAPDH monoclonal antibodies (1:2000, Sigma, St. Louis, MO, USA) overnight. Horseradish peroxidase-linked anti-mouse IgG was then added (1:15000, Sigma, St. Louis, MO, USA) and ECL visualization of the bands was performed as previously described [30].

### In vitro effects of N-Acetylcysteine on TNF- production

In total, 2 × 10^5 ^HFCL/EF, HFCL/F, HFCL/pCI and HFCL cells were seeded in 96-well plates. The cells were allowed to adhere to the plates and then washed with PBS three times. HFCL/EF cells were treated with 2, 100, or 200 mmol/L 5-FU in serum-free medium. The culture supernatant was collected after 24 h. Cells were treated with a combination of 20 mmol/L N-acetylcysteine (NAC; Sigma St. Louis, MO, USA) and various concentrations of 5-FU for 24 h. FL expression was measured using an ELISA kit (R&D, Minneapolis, MN, USA) according to the manufacturer's instructions.

### Statistical Analysis

All measurements were performed at least in triplicate. Statistical evaluation of the raw data was performed using one-way analysis of variance (ANOVA). Data are presented as mean ± standard deviation (SD).

## Results

### Effect of 5-FU treatment on EGFP expression in HFCL/EF

**Figure 1:**
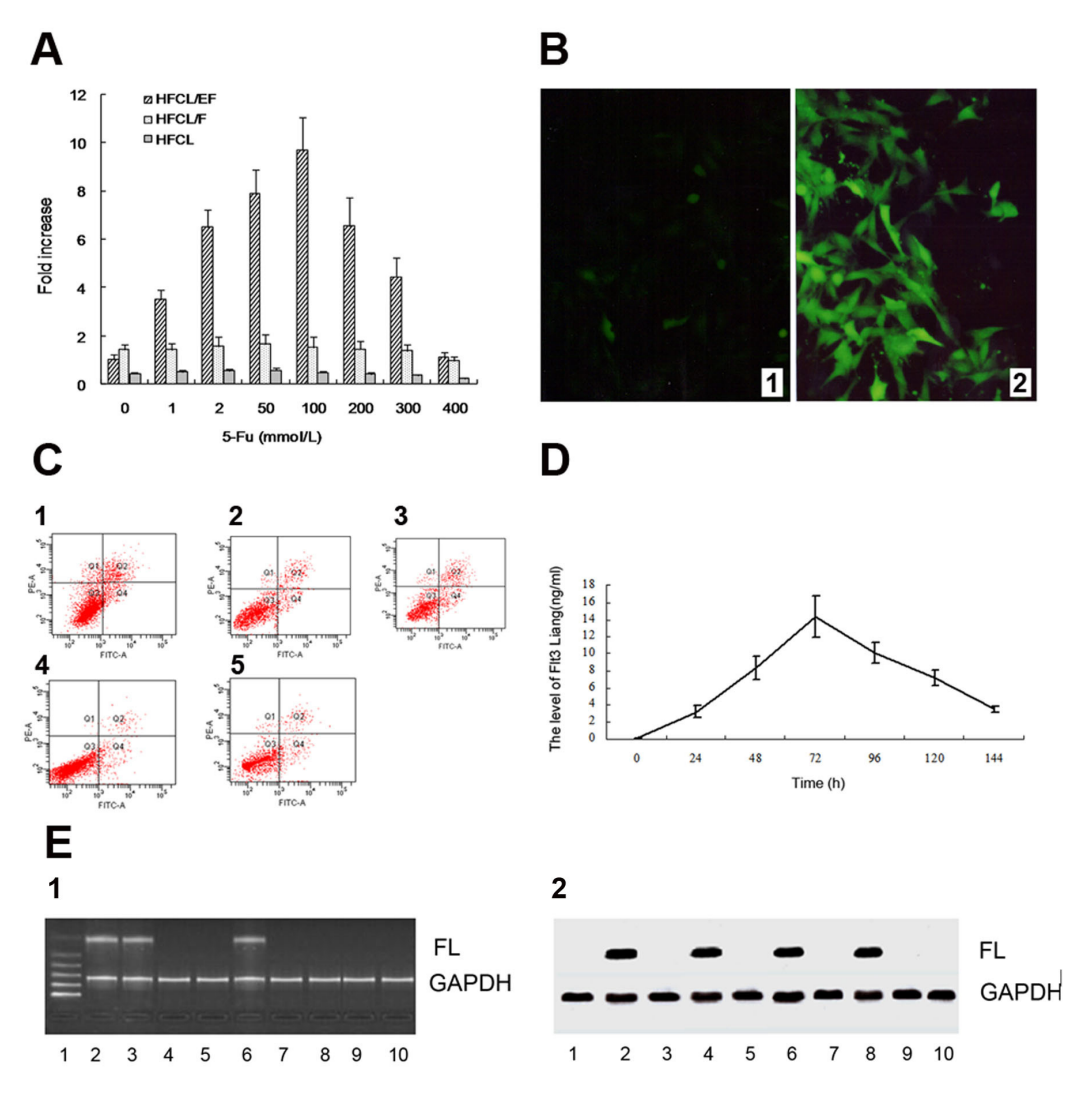
**A: Induced expression of EGFP in HFCL cells transfected with Egr-EF after exposure to 5-FU**. EGFP expression in HFCL/EF cells transfected with Egr-EF was used to evaluate activation of the Egr-1 promoter by 5-FU treatment. EGFP expression in HFCL/EF cells exposed to 1, 2, 50, 100, 200, 300 mmol/L 5-FU was significantly higher than that in HFCL/EF cells not exposed to 5-FU and the HFCL group, P < 0.01. However, at 400 mmol/L 5-FU, there was no difference between the 5-FU treated group and the untreated group. Data are reported as mean ± SEM: **B: EGFP expression confirmed by fluorescence inverse microscopy**. (1) HFCL/EF group × 400. (2) HFCL/EF + 5-FU group × 400. **C: Effect of supernatant from HFCL/EF cells treated with 5-FU on CD34^+ ^cells**. (1) HFCL/EF + 5-FU, (2) HFCL/EF, (3) HFCL/F + 5-FU, (4) HFCL/pCI + 5-FU, (5) HFCL + 5-FU. **D: FL expression by HFCL/EF cells after Egr-1 promoter activation with 5-FU**. FL production by HFCL/EF cells exposed to 5-FU (100 mmol/L) was measured using ELISA assay. Significant increases in FL protein levels were detected 24, 48, 72, 96, 120 and 144 h after exposure to 5-FU (P < 0.01, versus FL protein level at 0 h). Data are reported as mean ± SEM. **E: (1)RT-PCR analysis of FL mRNA levels in HFCL/EF cells after 5-FU treatment**. 1. 1.DNA Marker 11, 2.HFCL/EF with 5-Fu treatment group (in vitro), 3.HFCL/F without 5-Fu treatment group(in vitro), 4.HFCL/EF without 5-Fu treatment group(in vitro), 5.HFCL with 5-Fu treatment group (in vitro), 6.HFCL/EF plus 5-Fu group (in vivo), 7.HFCL/EF plus NS group(in vivo), 8. HFCL plus 5-Fu group (in vivo), 9. HFCL plus NS group(in vivo), 10. HFCL/pCI with 5-Fu treatment group (in vitro). **(2) Western blot analysis of FL protein levels in HFCL/EF cells after 5-FU treatment**. 1. 1. HFCL/EF without 5-Fu treatment group (in vitro), 2. HFCL/EF with 5-Fu treatment group (in vitro), 3. HFCL/pCI with 5-Fu treatment group (in vitro), 4. HFCL/F with 5-Fu treatment group (in vitro), 5. HFCL with 5-Fu treatment group (in vitro), 6. HFCL/F without 5-Fu treatment group (in vitro), 7. HFCL/EF plus NS group(in vivo), 8. HFCL/EF plus 5-Fu group(in vivo), 9. HFCL plus 5-Fu group(in vivo), 10. HFCL plus NS group(in vivo), Lain 2. the consistent expression of GAPDH in various groups.

EGFP expression in cultured HFCL/EF cells was higher in cells treated with 5-FU over a range of doses (0 - 400 mmol/L, Figure 1.A) than in those not treated with 5-FU. Increased EGFP expression indicates that the Egr-1 promoter could induce downstream gene expression after treatment with the chemotherapeutic agent 5-FU.

The fluorescent intensity of the group treated with 100 mmol/L 5-FU was five times higher than the group without 5-FU treatment. The enhancement of EGFP expression with 5-FU treatment declined with treatment above 100 mmol/L 5-FU. At 400 mmol/L 5-FU, there was no difference between the 5-FU treated group and the untreated group. In cells isolated from mice injected with HFCL/EF cells, treatment with 5-FU resulted in higher levels of EGFP expression than in the absence of 5-FU treatment (Figure 1.B). Both the HFCL + NS group and HFCL + 5-FU groups were negative for EGFP-expressing cells. The HFCL/EF + NS and HFCL/EF + 5-FU groups contained 0.12 ± 0.05% and 0.26 ± 0.08% EGFP-expressing cells, respectively.

### Effect of supernatants from 5-FU treated HFCL/EF cells on CD34^+ ^proliferation

Serum-free supernatants from 5-FU-treated HFCL/EF cells increased CD34^+ ^cellular proliferation (Figure 1.C). After 10 days of culture, the number of CD34^+ ^cells cultured with supernatant from HFCL/EF + 5-FU cells was 163.11 ± 10.58 × 10^3 ^cells/ml, which was significantly higher than that of the control groups (HFCL/EF: 69.01 ± 12.73 × 10^3 ^cells/ml; HFCL/pCI + 5-FU: 70.31 ± 15.02 × 10^3 ^cells/ml; HFCL/F + 5-FU: 93.56 ± 22.68 × 10^3 ^cells/ml; HFCL + 5-FU group: p < 0.01).

### FL secretion from HFCL/EF cells after 5-FU treatment

The FL content in serum-free supernatants from HFCL/EF cell culture was determined by ELISA assays. HFCL transfected cells did not produce detectable amounts of FL from endogenous FL genes. Supernatants from HFCL/EF cells contained 0.15 ng/ml FL. Supernatants from HFCL/EF cells treated with 5-FU (100 mmol/L) contained even higher FL levels. Maximum FL production by HFCL/EF cells treated with 5-FU was observed after 72 h (14.35 ng/ml, Figure 1.D). FL levels secreted by HFCL/EF cells were significantly higher than without 5-FU treatment at all time points (p < 0.01).

### RT-PCR analysis of FL mRNA levels in HFCL/EF cells treated with 5-FU

FL mRNA levels in 5-FU-treated HFCL/EF cells were measured by RT-PCR. FL mRNA levels in HFCL/EF cells were significantly higher in cells treated with 5-FU. FL mRNA was also detected in HFCL/F cells, but levels were low without 5-FU treatment (Figure 1.E.1). No FL mRNA was detected in the HFCL + NS, HFCL + 5-FU or HFCL/EF + NS groups. FL mRNA levels were higher in bone marrow cells from mice injected with HFCL/EF cells and treated with 5-FU.

### Western blot analysis of FL protein expression in HFCL/EF cells

FL protein expression was not detected in the HFCL cells cultured *in vitro *(Figure 1.E.2). Treatment of the HFCL/EF group with 5-FU resulted in significantly higher levels of FL protein expression than in the HFCL, HFCL/F, HFCL/pCI and HFCL/EF without 5-FU treatment groups. In the *in vivo *study, FL protein expression was detected in bone marrow cells of tumor-bearing mice treated with 5-FU and injected with HFCL/EF cells. No detectable FL expression was found in any control group (Figure 1.E.2, lanes 7, 9 and 10).

### Effect of N-acetylcysteine on FL secretion in HFCL/EF cells treated with 5-FU

**Figure 2:**
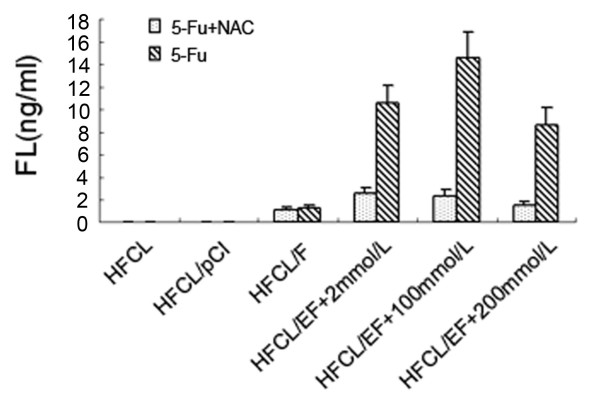
**Effect of N-acetylcysteine on FL gene expression in HFCL/EF cells treated with 5-FU**. FL expression of HFCL/EF cells exposed to 5-FU (2, 100, 200 mmol/L) with or without addition of N-acetylcysteine (200 mmol/L). FL protein levels secreted by HFCL, HFCL/pCI and HFCL/F cells are shown for comparison.

FL secretion in NAC-treated (200 mmol/L) 5-FU-treated (2, 100, 200 mmol/L) HFCL/EF cells was significantly less (P < 0.01) than for 5-FU-treated HFCL/EF cells not exposed to NAC (Figure 2). NAC had no effect on the HFCL/F group (*P *> 0.05). FL secretion in the HFCL and HFCL/pCI groups was not detectable (with or without NAC). In the HFCL/F group, there was no significant difference in FL secretion with or without NAC.

### Effect of 5-FU induced gene therapy on peripheral blood cell count in tumor-bearing mice

**Figure 3:**
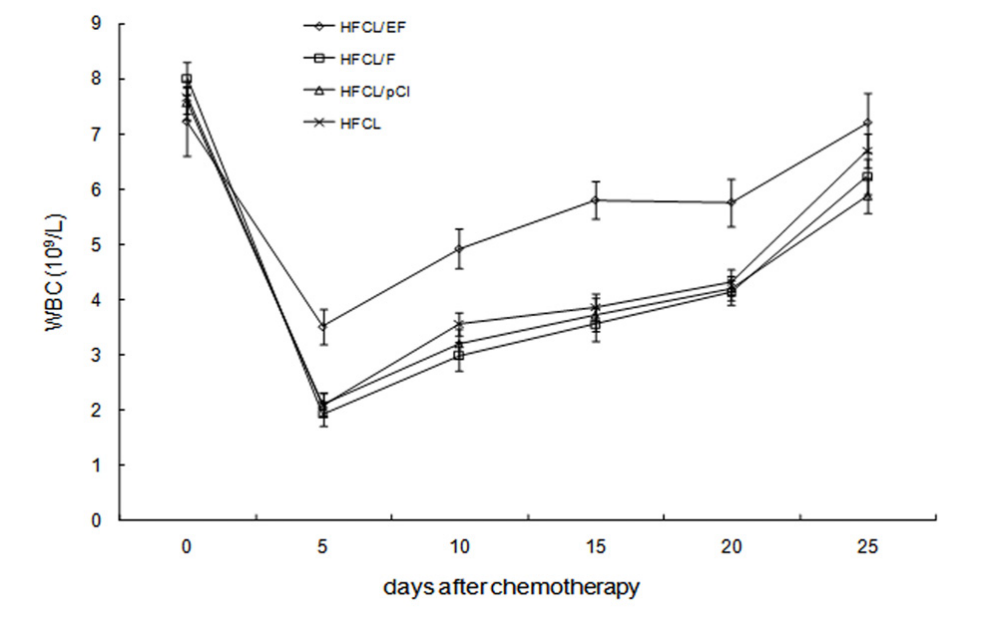
**Peripheral white blood cell counts in mice after 5-FU treatment**. Results are shown for mice injected with HFCL/EF, HFCL/F, HFCL/pCI and HFCL cells at days 0 through 25 (n = 6/group).

**Figure 4:**
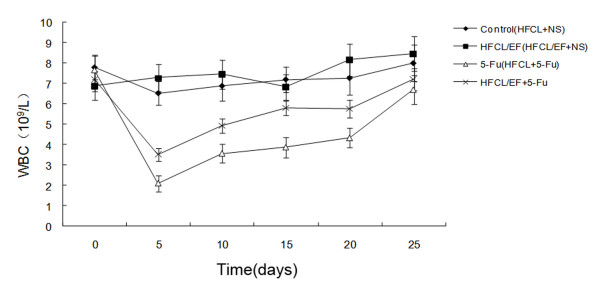
**Peripheral white blood cell counts in mice after 5-FU or saline**. **Treatment**. Results are shown for HFCL/EF + 5-FU, HFCL + 5-FU, HFCL/EF + NS and HFCL + NS at days 0 through 25 (n = 6/group).

All mice were inoculated by B16 cells, and black spots were gradually seen in BALB/c mice's back skin, feet or ankle 5 days later. Among them, the symptoms such as, fast tumor growth, ulceration and hemorrhage were observed in one mouse, which died at 15 days following the inoculation. All but one mouse (HFCL group) survived the 25 days of observation. Blood was withdrawn from the tail vein every week and used to determine white blood cell counts, platelet counts and Hemoglobin levels. The white blood cell (WBC) counts of the HFCL/EF group 5, 10, 15 and 20 days after 5-FU treatment (100 mg/kg) were higher than those for the HFCL, HFCL/pCI, and HFCL/F groups (P < 0.05, Figure 3). The WBC count of the HFCL/EF group was 3.51 ± 0.62 × 10^9^/L compared to 4.92 ± 0.72 × 10^9^/L, 5.80 ± 0.68 × 10^9^/L, 5.76 ± 0.86 × 10^9^/L for the with HFCL, HFCL/pCI and HFCL/F groups, respectively. In addition, the hematopoietic recovery of the HFCL/EF group was faster than the HFCL, HFCL/pCI and HFCL/F groups. Hemoglobin (Hb) levels and platelet (PLT) counts were not significantly different among the four groups. The WBC counts for the HFCL/EF + NS and HFCL + NS groups did not change significantly over 0 to 20 days (Figure 4). Treatment with 5-FU resulted in decreased WBC counts in both the HFCL/EF + 5-FU and HFCL + 5-FU groups. However, the WBC count of the HFCL + 5-FU group was lower than that of the HFCL/EF+5-FU group. Periodic analyses of peripheral blood cells from tumor-bearing mice showed an increase in the number of white blood cells at an early stage after chemotherapy.

### Effect of 5-FU-mediated hematopoietic growth factor gene therapy on tumor size

**Figure 5:**
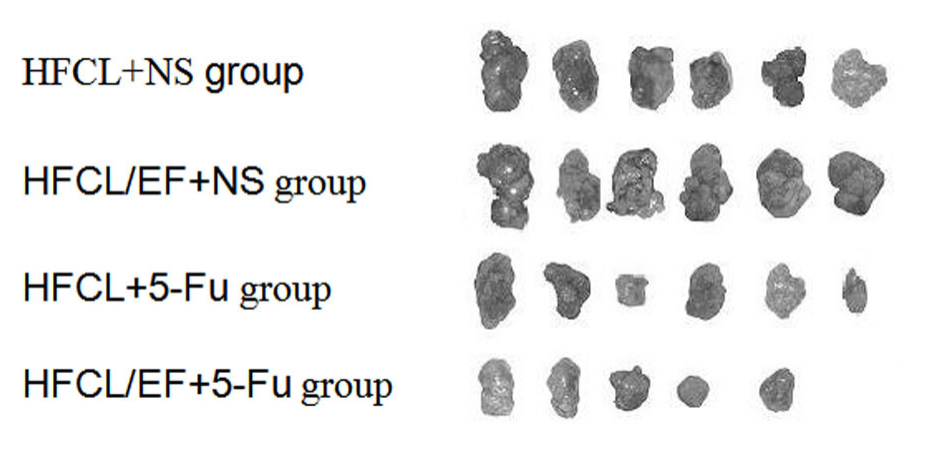
**Effect of 5-FU-mediated hematopoietic growth factor gene therapy on tumor size**. By day 25, tumor volumes were 1422.61 ± 320.32 mm^3 ^(HFCL + 5-FU) and 1282.35 ± 451.15 mm^3 ^(HFCL/EF + 5-FU), and the tumor inhibitory rates were 46.20% and 51.51%, respectively. The tumor volumes in the HFCL + NS and HFCL/EF + NS group increased from 0 to 25 days, reaching volumes of 2644.43 ± 466.12 mm^3 ^and 2480.54 ± 688.24 mm^3^, respectively. The corresponding inhibitory rates were 0.00 and 0.06%, respectively.

After 10 - 12 days, reductions were observed in tumor volumes in the HFCL + 5-FU and HFCL/EF + 5-FU groups (Figure 5). By day 25, tumor volumes were 1422.61 ± 320.32 mm^3 ^(HFCL + 5-FU) and 1282.35 ± 451.15 mm^3 ^(HFCL/EF + 5-FU), and the tumor inhibitory rates were 46.20% and 51.51%, respectively. The tumor volumes in the HFCL + NS and HFCL/EF + NS group increased from 0 to 25 days, reaching volumes of 2644.43 ± 466.12 mm^3 ^and 2480.54 ± 688.24 mm^3^, respectively. The corresponding inhibitory rates were 0.00 and 0.06%, respectively.

## Discussion

Egr-1 gene expression is induced by a variety of stimuli, including growth factors, IR, chemotherapy, hypoxic stress and ROIs [34]. Transcriptional activation of Egr-1 requires the CArG DNA sequence element in the promoter region of this gene [35]. Radiation-gene therapy using TNFerade, a second-generation E1-, partial E3- and E4-deleted adenoviral vector carrying the transgene encoding human TNF-a downstream of the Egr-1 promoter, has been used in two phase I trials for patients with various solid tumors [36]. IR has been used to produce ROIs that activate the highly conserved sequence CArG of the Egr-1 promoter, thereby regulating the expression of downstream therapeutic target genes [17,18,37].

We have previously constructed a pCI-neo expression vector containing the *Egr-1 *regulatory sequence including the CArG element upstream from FL cDNA to promote FL expression. IR was used to activate the Egr-1 promoter to regulate FL gene expression in human bone marrow stromal cells injected into SCID mice.

Hematopoietic recovery was promoted by IR activation of FL expression. Chemotherapy-induced and target-regulated gene therapy is a new approach for tumor chemotherapy and gene therapy [38]. Chemotherapeuticdrugs significantly injure hematopoietic tissues. Therefore, combining chemotherapy and hematopoietic growth factors in gene therapy is one approach to reduce the damage caused by chemotherapy. 5-FU is a commonly used, broad-spectrum anti-tumor drug that causes tumor cell death by producing ROIs that result in DNA damage. The half-life of 5-FU in mice and humans is only 0.5 h, and 5-FU affects proliferative cells, especially those in S phase. Its principal restrictive toxicity is marrow hematopoietic depression [39]. Iko et al. [40] found that reactive oxygen species limit the productive lifespan of hematopoietic stem cells. Flt3 Ligand (FL) is a cytokine that regulates early hematopoiesis by regulating the proliferation and differentiation of early hematopoietic stem cells and progenitor cells, as well as enhancing hematopoietic and immunological functions [41]. Bone marrow stromal cells are the ideal cell type targets for gene therapy, as these cells determine the speed of hematopoietic function recovery after chemotherapy [42]. We hypothesized that ROS produced by 5-FU treatment could be used activate the Egr-1 promoter and thereby regulate downstream FL gene expression. Promotion of FL gene expression should reduce hematopoietic injury caused by chemotherapy. In this study, EGFP and FL cDNA were inserted into the eukaryotic expression vector pCI-neo containing the Egr-1 promoter. The vector was transfected into bone marrow stromal cells (HFCL/EF). The integration and expression of the exogenous EGFP gene was confirmed by expression of green fluorescent protein in HFCL/EF cells detected by FACS. FL expression was confirmed by RT-PCR detection of FL mRNA and detection of FL protein by western blot and ELISA assays.

Chemo-inducible FL gene therapy was demonstrated by hemotherapy-induced activation of the Egr-1 promoter resulting in enhanced recovery from hematopoietic injury. However, the proper dose of 5-FU for this hematopoietic factor gene therapy approach still needs to be determined.

The oxygen free radical inhibitor NAC was used to block ROI formation in order to verify whether 5-FU induces FL expression through ROI generation. FL expression was significantly decreased in the presence of NAC, indicating that oxygen free radical production is involved in activation of the Egr-1 promoter. Oxygen free radicals are produced by a number of chemotherapeutic drugs [43,44]. Consequently, other chemotherapeutic drugs could also be used for gene therapy using the Egr-1 promoter.

In our study, 5-FU was employed to activate the Egr-1 promoter in human bone marrow stromal cells injected into tumor-bearing mice, resulting in exogenous FL expression. EGFP positive cells were observed by flow cytometry and fluorescence microscopy, indicating that the transplanted human HFCL/EF cells were viable. EGFP expression increased in the HFCL/EF group after 5-FU treatment, indicating that 5-FU activated the Egr-1 promoter, which resulted in downstream gene expression.

These results are similar to those observed by Lopez [23]. In addition, RT-PCR and western blot assays demonstrated that human FL mRNA and protein are expressed in bone marrow cells after 5-FU treatment. FL mRNA and protein levels were not significantly different between the HFCL/EF + 5-FU, HFCL/EF and HFCL/F groups. This may be due to hematopoietic function recovery that occurred over the 25 days after chemotherapy.

Hou et al. [45] Combined FL gene therapy with 5-FU treatment and observed a synergistic effect on tumor therapy. In our study, 5-FU-induced FL gene therapy showed no effect on the growth of transplantation tumors. The tumor inhibitory rate was only related to the chemotherapy group, indicating that FL expression does not directly affect the efficacy of chemotherapy on tumor reduction. The decline of peripheral white blood cells in the HFCL/EF + 5-FU group was less than that in the control group. The hematopoietic absence in the agranulocytosis stage was also shortened in the HFCL + 5-FU group versus the control group.

Recent research has indicated that targeted gene therapy mediated by activation of the Egr-1 promoter with chemotherapeutic drugs is a promising treatment option for solid tumors [46]. In this study, chemotherapy was used to activate the Egr-1 promoter, resulting in expression of a hematopoietic growth factor that reduced hematopoietic injury. The hematopoietic growth factor did not affect tumor growth, but it reduced the hematopoietic injury associated with chemotherapy. Since the Egr-1 promoter is activated by ROIs, it can be used to induce expression of downstream hematopoietic growth factors during chemotherapy, thereby improving hematopoietic recovery. Gene therapy using expression of the tumor necrosis factor regulated by the Egr-1 promoter has already been used in humans for tumor therapy. Hematopoietic factor gene therapy regulated by chemotherapy-induced activation of the Egr-1promoter shows great promise [29]. In the future, other genes could be used in chemotherapy-induced gene therapy for various diseases.

## Competing interests

The authors declare that they have no competing interests.

## Authors' contributions

ND carried out the molecular genetic studies, drafted the manuscript. XP analyzed with specific molecular markers on the transcription and protein expression level. JZ carried out the immunoassays. HZ participated in study of expression of RNA and DNA and protein structures. YF participated in the design of the study and performed the statistical analysis. YH participated in its design and coordination and helped to draft the manuscript. All authors read and approved the final manuscript.

## Acknowledgements

This work was supported by Chinese National Key project of Basic Research (No.1999053903), the National Natural Science Foundation of China (Both Transcriptional and Translational Targeting Regulation Strategies for Transcriptional Factor Inducied by Chemotherapy in Melanoma Stem Cells. Grant No.39900040), and the National Natural Science Outstanding Youth Foundation of China (Grant No.39825111). The authors gratefully acknowledge Dr. Lu Xing and Dr Li Liang (Institute of Radiation Medicine, Beijing, China), Dr. Wang Lisheng (Indiana University, Indianapolis, IN, USA), and Dr. Ye Chuanzhong (Vanderbit Epidemiology Center Institute for Medical Center North, Nashville, USA) for their helpful advice.
